# Metformin Treatment Is Associated with a Decreased Risk of Nonproliferative Diabetic Retinopathy in Patients with Type 2 Diabetes Mellitus: A Population-Based Cohort Study

**DOI:** 10.1155/2020/9161039

**Published:** 2020-04-19

**Authors:** Yu-Pei Fan, Chien-Tung Wu, Jiun-Lu Lin, Chao A. Hsiung, Hsiao Yu Liu, Jung-Nien Lai, Chen-Chang Yang

**Affiliations:** ^1^Institute of Public Health, National Yang-Ming University, Taipei, Taiwan; ^2^Department of Medical Education, MacKay Memorial Hospital, Taipei, Taiwan; ^3^Institute of Traditional Medicine, School of Medicine, National Yang-Ming University, Taipei, Taiwan; ^4^Department of Chinese Medicine, Taipei City Hospital, Linsen Chinese Medicine Branch, Taipei, Taiwan; ^5^Taiwan Association for Traditional Chinese Medicine of Family, Taipei, Taiwan; ^6^Division of Endocrinology and Metabolism, Department of Internal Medicine, MacKay Memorial Hospital, Taipei, Taiwan; ^7^Institute of Population Health Sciences, National Health Research Institutes, Taiwan; ^8^School of Chinese Medicine, College of Chinese Medicine, China Medical University, Taichung, Taiwan; ^9^Departments of Chinese Medicine, China Medical University Hospital, Taichung, Taiwan; ^10^Institute of Environmental and Occupational Health Sciences, National Yang-Ming University School of Medicine, Taipei, Taiwan; ^11^Division of Clinical Toxicology and Occupational Medicine, Department of Medicine, Taipei Veterans General Hospital, Taipei, Taiwan

## Abstract

**Purpose:**

To assess the relationship between metformin use and the severity of diabetic retinopathy (DR) in patients with type 2 diabetes mellitus (T2DM) and to investigate the effect of metformin dosage on reducing the incidence of DR.

**Methods:**

The study population included patients with newly diagnosed T2DM, who were aged ≥20 years and prescribed with antidiabetic drug therapy lasting ≥90 days, as identified using the National Health Insurance Research Database between 2000 and 2012. We matched metformin users and nonusers by a propensity score. Cox proportional hazard regression analyses were used to compute and compare the risk of developing nonproliferative diabetic retinopathy (NPDR) in metformin users and nonusers.

**Results:**

Overall, 10,044 T2DM patients were enrolled. Metformin treatment was associated with a lower risk of NPDR (aHR 0.76, 95% CI 0.68–0.87) and sight-threatening diabetic retinopathy (STDR, aHR 0.29, 95% CI 0.19–0.45); however, the reduction in risk was borderline significant for STDR progression among NPDR patients (aHR 0.54, 95% CI 0.28–1.01). Combination therapy of metformin and DPP-4i exhibited a stronger but inverse relationship with NPDR development (aHR 0.32, 95% CI 0.25–0.41), especially at early (<3 months) stages of metformin prescription. These inverse relationships were also evident at different metformin doses and in adapted Diabetes Complications Severity Index scores (aDCSI). Moreover, combination therapy of metformin with sulfonylureas was associated with an increased risk of NPDR.

**Conclusion:**

Metformin treatment in patients with T2DM was associated with a reduced risk of NPDR, and a potential trend was found for a reduced STDR risk in patients who had previously been diagnosed with NPDR. Combining metformin with DPP-4i seemingly had a significantly beneficial effect against NPDR risk, particularly when aDCSI scores were low, and when metformin was prescribed early after T2DM diagnosis. These results may recommend metformin for early treatment of T2DM.

## 1. Introduction

Diabetic retinopathy (DR) is one of the common microvascular complications in patients with type 2 diabetes mellitus (T2DM), characterized by microscopic, blood-filled, arterial wall bulges. These bulges usually do not produce noticeable symptoms at initial stages and are identified as nonproliferative diabetic retinopathy (NPDR) [1]. As the disease progresses, tiny spots or blood clots may accumulate in the retina, resulting in retinal ischemia and driving progression to a sight-threatening diabetic retinopathy (STDR), which is the major cause of blindness among the working-age population around the world [2, 3]. Of note, the annual incidence of DR ranges from 2.2% to 12.7% and progression to proliferative DR from 3.4% to 12.3% [4], despite the recent improvements in the systemic treatment of metabolic disorders and the common use of applied laser photocoagulation.

Good glycemic control remains the core foundation of managing T2DM. Pharmacotherapy plays a vital role in preventing or delaying the onset and progression of the irreversible microvascular complications of T2DM, such as damage related to retinopathy and nephropathy [5–7]. The major classes of oral antidiabetic medication include biguanides (e.g., metformin), sulfonylureas, meglitinide, thiazolidinedione (TZD), dipeptidyl peptidase 4 (DPP-4) inhibitors, and *α*-glucosidase inhibitors (e.g., acarbose). These medications improve insulin sensitivity, stimulate insulin production by the pancreatic beta cells, slow down the intestinal absorption of ingested carbohydrates, and strengthen the action of intestinal hormones (incretins) involved in controlling blood sugar [5, 8]. Besides its glucose-lowering effects, metformin, a first-line treatment for T2DM, has considerable antiangiogenic and anti-inflammatory effects on the retinal vasculature, as shown by animal studies [9–11]. Furthermore, metformin reduces the proliferation of retinal epithelial cells, according to a small prospective pilot clinical trial [12]. These observations suggest that metformin exerts beneficial effects that ameliorate microvascular complications. However, not all antidiabetic drugs exhibit effects similar to those of metformin. For example, a recent meta-analysis showed an increased risk of developing DR among subjects treated with sulfonylureas, compared to subjects receiving placebos. The effects of other antidiabetic drugs, such as thiazolidinediones, DPP-4i, glucagon-like peptide-1 receptor agonist (GLP-1RA), or sodium-glucose cotransporter 2 (SGLT2) inhibitors on the risk of developing DR, remain uncertain in patients with T2DM [13].

As noted previously, combination therapy with metformin plus a second agent may help patients achieve HbA1c targets more quickly than monotherapy [14, 15]. Unfortunately, there has been no detailed analysis of the effects of these metformin combination therapies on the risk of developing DR. Also, the most effective dose of metformin for such effects has not been established. Hence, the major aim of the present study was to assess the effect of metformin (mono- or multitherapy) on the severity of DR, including NPDR and STDR in patients with T2DM. Furthermore, we examined the effect of metformin dose on reducing the risk of DR.

## 2. Method

### 2.1. Data Source

This study was conducted by employing the claims data obtained from the Longitudinal Health Insurance Database (LHID), a representative subset of the National Health Insurance Research Database (NHIRD) in Taiwan. The LHID comprises information on one million National Health Insurance (NHI) beneficiaries in Taiwan, sampled at random from the population-wide NHI registry based on the population in the year 2000 and covered all claims data annually between 1996 and 2012. The disease records were based on the International Classification of Diseases, Ninth Revision, Clinical Modification (ICD-9-CM). The present study used both inpatient and outpatient claims data from 2000 to 2012. The Research Ethics Committee of China Medical University and Hospital approved this study (approval number: CMUH105-REC3-015).

### 2.2. Study Population

**Figure 1:**
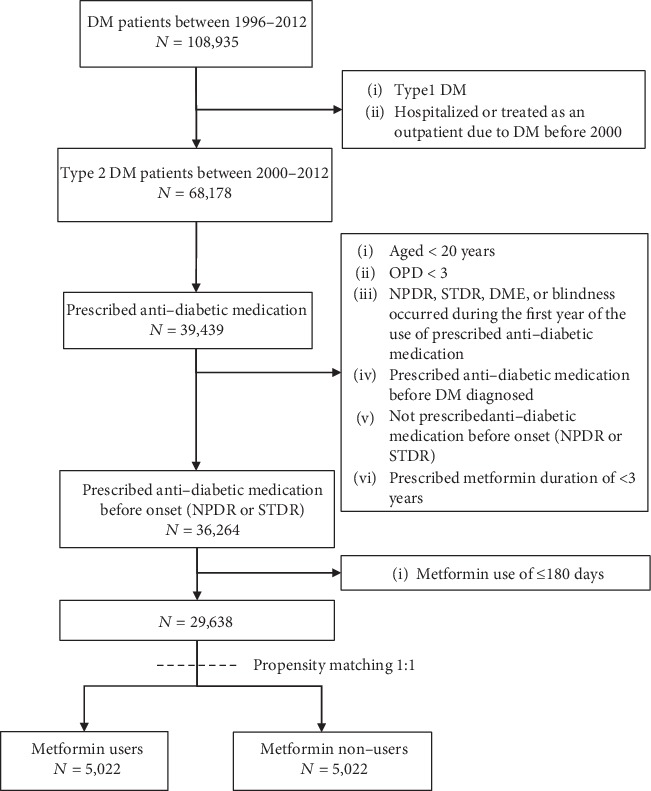
Study flow chart.

In this study, we compared the risk of newly diagnosed DR between T2DM patients with and without metformin therapy between January 1, 2000 and December 31, 2012. For each enrolled patient, the index date was the date of the first prescription of any antidiabetic drug. Subjects eligible for this study were selected from the NHIRD and met the following criteria during the study period: (i) age of ≥20 years; (ii) presence of T2DM, based on the record of any hospital admission with a diagnostic code of T2DM (ICD-9-CM 250) and/or three or more outpatient visits with a T2DM diagnostic code within 365 calendar days; and (iii) receipt of a prescription of antidiabetic drugs for a period exceeding 90 days. Patients diagnosed with type 1 diabetes mellitus (ICD-9-CM code 250.x3), those not prescribed with antidiabetic medication before DR diagnosis, those prescribed antidiabetic drugs before T2DM diagnosis, and those prescribed metformin for periods ≤ 180 days were excluded from further analysis. Patients diagnosed with NPDR, STDR, diabetic macular edema, and blindness during the first year of the antidiabetic drug prescription were also excluded to avoid misclassifying individuals with retinal diseases unrelated to antidiabetic drug treatment (Figure 1).

### 2.3. Definition of Outcomes (NPDR and STDR)

The examined outcome in this study was DR, including NPDR and STDR. We followed the enrolled patients until the end of 2012, unless they had been diagnosed with DR earlier, or could be considered lost to follow-up because of death, withdrawal from the database, or other reasons. The grading of DR severity and the presence of clinically significant macular edema were determined by a retinal specialist, according to the definitions of Early Treatment Diabetic Retinopathy Study (ETDRS; [16]). Accordingly, patients were classified with either mild or moderate NPDR or SNPDR/PDR. Codes for photocoagulation treatment were also determined according to the guidelines derived from the ETDRS [17] and the Diabetic Retinopathy Study [18]. In addition, we classified patients with DR as having NPDR and STDR. NPDR cases were identified by an ICD code (ICD-9-CM 250.5, 362.01, 362.03–06 362.1, 362.81, and 362.82). Patients with STDR were classified according to their first exposure to any of the following NHI procedures: pan-retinal photocoagulation (codes: 60003C and 60004C), macular photocoagulation (60001C and 60002C), and pars plana vitrectomy (86206B and 86207B).

### 2.4. Potential Confounding Factors

We identified potential confounders that might influence the development or progression of DR, i.e., age, gender, comorbidities, medications, and severity of T2DM. Existing comorbidity was noted when a patient had been diagnosed with the comorbidity before the index date. Comorbidities included in this study were hypertension (401–405), cerebrovascular accidents (390–420 or 431–438), diabetic polyneuropathy (3572, 24960, 24961), diabetic nephropathy (2494, 2504), and dyslipidemia (272). Considered medications were antihypertensive drugs and antihyperlipidemic drugs that were prescribed before the index date. The severity classification of T2DM was based on scores according to the adapted Diabetes Complications Severity Index (aDCSI). The aDCSI incorporates a uniquely wide range of diabetic complications and is a useful tool for predicting mortality and hospitalizations among T2DM patients. It may be used to adjust for the baseline severity of disease in the T2DM population [19, 20]. The aDCSI was validated using the NHIRD, and its power to predict the risk of hospitalization for T2DM patients in the NHIRD was similar to that in the original study [21].

### 2.5. Statistical Analysis

We expected metformin users to be different from metformin nonusers with respect to certain important prognostic factors, which might confound the outcome analyses. Propensity score matching, a method that is aimed at reducing the selection bias in nonexperimental, nonrandomized, and retrospective observational studies, was thus employed in this study. We matched each patient in the metformin-user cohort with someone who had similar baseline characteristics such as age, gender, comorbidities, medication use, aDCSI scores, and DM duration before taking antidiabetic drugs in the metformin nonuser cohort. Based on the balancing guidelines [22], we selected 1 : 1 propensity score-matched samples of metformin users and metformin nonusers that had balanced baseline characteristics.

We used Cox proportional hazard regression analyses to compare the risk of developing NPDR in metformin users and nonusers. Furthermore, to assess dose effects of metformin, we conducted subgroup analyses in prespecified strata of the cumulative defined daily dose (DDD) (≤360 DDDs, 361–720 DDDs, or >720 DDDs), and all information on metformin prescriptions was extracted from the NHI prescription database. We collected the date of prescription, daily dose, and the number of days that the drug was prescribed. DDD, recommended by the World Health Organization of 2,000 mg/day, was used to quantify the dose of metformin treatment. For each patient, the cumulative dosage of metformin, expressed as the number of DDDs, was calculated based on all of the prescriptions dispensed during the follow-up period.

## 3. Results

Among the entire study cohort of 29,638 patients diagnosed with T2DM and receiving antidiabetic medication prescription between Jan 1, 2000 and Dec 31, 2012 (Figure 1), 24,611 individuals used metformin and 5,027 were nonusers. Before matching, several differences were noted between the two groups of patients (Table 1). Metformin users were younger, had suffered from T2DM for a shorter period between diagnosis and prescription of antidiabetic medications, had lower aDCSI scores, and were less likely to receive antihypertensive drugs and antihyperlipidemic drugs compared with unmatched nonusers of metformin. After matching participants in a 1 : 1 ratio according to the propensity score, 5,022 patients were included in the primary outcome analysis as metformin users and nonusers. The two matched groups of patients were similar with respect to all covariates (Table 1).

Among the T2DM patients, metformin users were less likely to develop NPDR and STDR than nonusers. The adjusted hazard ratio (aHR) was 0.76 (95% CI 0.68–0.87) for NPDR development and 0.29 (95% CI 0.19–0.45) for STDR development (Table 2). The dose-response analysis showed a decreased NPDR risk in patients who received a cumulative dose of metformin > 360 DDDs. Patients who received >1,440 DDDs showed the greatest reduction in the risk of developing NPDR among metformin users (adjusted HR 0.29, 95% CI 0.19–0.43; *p* = 0.01 for trend analysis) (Table 3). The estimated dose-response effect of metformin use on STDR showed the same pattern (Supplementary Table 1). We also analyzed whether metformin could be associated with an inverse relation in the progression to STDR among NPDR patients. However, no significant difference was found between metformin users and nonusers (adjusted HR 0.54, 95% CI 0.28–1.01) (Table 2).

**Figure 2:**
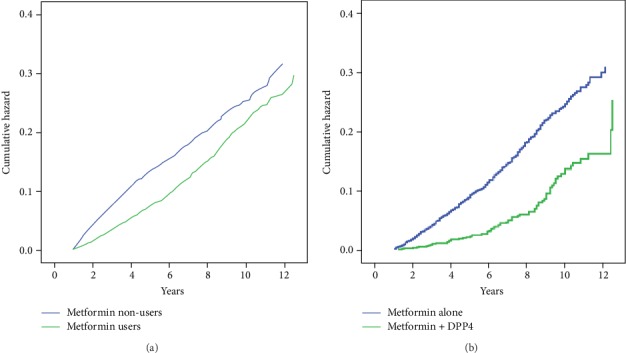
(a) Cumulative hazard of NPDR: comparison between metformin users and nonusers with DM. (b) Cumulative hazard of NPDR: comparison between metformin alone users and metformin + DPP-4i users with DM.

To assess the effects of antidiabetic drugs against NPDR, we further performed a multivariate analysis in all T2DM patients (Table 4 and Figure 2). We found that both metformin and DPP-4 inhibitors (DPP-4i) were associated with a significantly lower risk of NPDR (aHR 0.76, 95% CI 0.67–0.86 for metformin, aHR 0.36, 95% CI 0.22–0.60 for DPP-4i, model 4 in Table 4). Moreover, the inverse relation was stronger in T2DM patients treated with both drugs (aHR 0.32, 95% CI 0.25–0.41). In contrast, combined therapy with metformin and sulfonylureas did not reduce the risk of NPDR significantly (aHR 1.02, 95% CI 0.75–1.39) (Table 4).

We also analyzed the various subgroups of metformin users in more details. Given the notable differences between metformin treatment either as monotherapy or as a part of a combination therapy with DPP-4i, we evaluated the association of different duration and dosage of metformin therapy with NPDR occurrence. The subgroup analysis was stratified by time to metformin therapy after T2DM diagnosis. Compared with patients who received metformin monotherapy, those who were prescribed combination therapy had a significantly decreased NPDR risk in the early-prescription (<3 months) subgroup. Moreover, the seemingly beneficial effect occurred, to a different extent, in different metformin dosage groups (adjusted HR 0.38, 95% CI 0.21–0.68 for ≤360 DDDs; adjusted HR 0.36, 95% CI 0.19–0.69 for 361–720 DDDs; and adjusted HR 0.50, 95% CI 0.30–0.83 for >720 DDDs). The effect also varied by different aDCSI score groups (adjusted HR 0.36, 95% CI 0.24–0.55 for aDCSI = 0; adjusted HR 0.23, 95% CI 0.09–0.58 for aDCSI = 1; and adjusted HR 0.38, 95% CI 0.16–0.89 for aDCSI ≥ 2) (Table 5). There was a similar association when we included the DDDs of patients receiving combination therapy with DPP-4i into our analysis (Supplementary Table 2).

## 4. Discussion

The present study demonstrated an inverse dose-response relationship between the use of metformin monotherapy and the risk of NPDR in patients with T2DM. The effect remained after controlling for potential confounders. Moreover, there was an even greater reduction in the risk of NPDR among patients who received combination treatment with metformin and DPP-4i. A previous retrospective review of medical records of 234 patients with T2DM indicated that metformin prescriptions might be associated with protective effects against ocular complications, especially against DR [23]. In another medical-record review study, presented at the 2014 ARVO annual meeting, 333 patients with T2DM were monitored for more than 15 years. The results showed that 47.2% of patients from the nonmetformin-treated group developed severe DR requiring pan-retinal photocoagulation, compared to 25.1% of the patients who were treated with metformin (*p* < 0.001) [24]. Animal studies also indicated metformin's protective effects against DR [9, 10, 25]. The results of this population-based cohort study are consistent with those of previous studies. In addition, the seemingly beneficial effect of metformin on DR appears to work in the early stage rather than in the late stage of T2DM, which is a novel observation.

The present study included T2DM patients who were diagnosed by qualified physicians between 1999 and 2012, as recorded by a nationwide insurance database in Taiwan. Hence, we can rule out the possibility of selection bias introduced by nonrepresentative, small-scale samples. Additionally, data on antidiabetic drug usage were obtained from a fee-for-service healthcare claims database that covered all available prescription drugs from nearly all outpatient clinics and hospitals in Taiwan before the date of DR onset. This enabled us to calculate the cumulative dose accurately and to rule out the possibility of recall bias. Moreover, we allowed a latency period of one year before the diagnosis of DR to exclude the possibility of DR occurrence that was not related to the prescription of antidiabetic medication. Therefore, we conclude that an inverse dose-response relationship exists between the administered dose of metformin and the risk of NPDR.

The observed cumulative incidence rates in our study, i.e., 21.9 per 1,000 person years for NPDR and 2.1 for STDR, were lower than those found in previous studies on Asian populations [26, 27]. This was likely caused by the stricter case selection criteria, which were applied to increase the validity of our study. We only included patients who were prescribed antidiabetic drugs for more than 90 days and who received metformin for more than 180 days. Patients diagnosed with visual-related diseases during the first year after being prescribed antidiabetic drugs were also excluded to avoid outcome misclassification. Similar to previous studies, the present study also showed that metformin users had a reduced risk of developing STDR [24, 28]. We examined the NPDR subgroup more precisely to identify the possible association between antidiabetic drug use and the risk of progression to STDR. Patients with NPDR who received metformin showed a lower incidence of STDR; although the statistical significance was borderline (adjusted HR = 0.54, 95% CI: 0.28–1.01, *p* = 0.0545). We inferred that the effect of metformin might be protective mainly against NPDR occurrence and potentially against the progression of DR. Future prospective studies are warranted to better understand the ways in which metformin may affect DR progression in T2DM patients.

Our study showed a 24% reduced risk of retinopathy in T2DM patients treated with metformin. The results of a subgroup analysis underscored these findings by demonstrating a reduced risk of NPDR starting in patients who received 361–720 DDDs (adjusted HR 0.77, 95% CI 0.63–0.93, *p* = 0.0074) and reaching its pinnacle in those receiving more than 1,440 DDDs. Despite metformin's importance in reducing the risk of DR, little is known about the dose-dependent effect of metformin in these patient populations. Cumulating evidence from animal studies revealed that metformin could effectively protect endothelial cells via antiangiogenic and anti-inflammatory mechanisms [29, 30]. Metformin seems to exert its antiproliferative effects through the activation of AMPK, an intracellular regulator of energy metabolism. The activation of AMPK pathways results in the arrest of cellular growth and proliferation. Han et al. used different in vitro and in vivo models of either angiogenesis or inflammation to study metformin's effects [11]. Their results showed that metformin dose-dependently inhibited the proliferation of human retinal vascular endothelial cells (hRVECs) and likewise activated AMPK signaling pathways. This is in line with our findings of a strong inverse relationship between metformin use and the risk of NPDR and the absence of specific inhibitory effects of metformin therapy on the progression from NPDR to STDR.

There is a common agreement that the risk of developing DR or other microvascular complications of T2DM depends strongly on the duration and severity of hyperglycemia [31–33]. Treatment with DPP-4i may reduce microvascular complications [34] to a larger extent than treatment with sulfonylureas [35] and may normalize retinal capillary flow in T2DM patients [36]. Furthermore, our findings indicate that, when controlling for other confounding variables, a reduced NPDR risk was observed in patients taking metformin combined with DPP-4i relative to those receiving metformin monotherapy. Moreover, treatment with DPP-4i was an independent protective factor against DR progression, besides their effects to improve glycemic control [37]. There have been no direct clinical studies regarding DR progression following DDP-4i therapy, although saxagliptin seemed to normalize retinal capillary flow in T2DM patients without DR [36]. Several animal studies found that treatment with DPP-4i reduced blood-retinal barrier changes through a mechanism independent of enhanced insulin secretion and decreased inflammatory state and apoptosis in diabetic retinas [38–40]. In clinical settings, a small double-blind, placebo-controlled, crossover trial in 50 T2DM patients without retinopathy found that six weeks of treatment with DPP-4i significantly reduced retinal capillary blood flow and improved vasodilation [36]. These findings suggest that DPP-4i added to metformin might halt DR progression [34]. Since the time to metformin therapy to restore glycemic control following the diagnosis of T2DM is a critical factor in slowing DR progression, earlier introduction of metformin with DPP-4i seems to be appropriate.

Evidence from a network meta-analysis suggests that treatment with sulfonylureas may be associated with an increased risk of DR [13]. Similarly, we found that sulfonylurea treatment was associated with a higher risk of developing NPDR (aHR 1.30, 95% CI 1.05–1.61). Further analysis showed that the adjusted HR of metformin plus sulfonylurea combination treatment tended to be higher than that of other antidiabetic medications, but the difference was not statistically significant (Table 4). Given that the combination of metformin and sulfonylureas is the most commonly used oral combination therapy in the NHIRD, which is prescribed to two out of five T2DM patients receiving combined therapy, any detrimental effect of the combination therapy among T2DM patients would have a substantial influence. Further well-designed prospective studies are warranted to achieve a full understanding of the mechanisms of combined therapy with metformin and sulfonylureas.

Metformin is considered the preferred first-line antidiabetic pharmacotherapy unless it is not tolerated or is contraindicated. Patients with the strongest concerns about medication harmfulness tended to underuse antidiabetic drugs [41] because they may want to try lifestyle modifications first to see if any improvements in their control of T2DM can be made before starting medications. It also might be a difficult decision for the primary care doctors if—and when—the diabetic patient needs to start metformin during the early stages of diabetes management [42]. The findings from our study provide patients and primary care doctors with supportive evidence for an early start of antidiabetic medication to affect the course of the disease and improve the prognosis. Our subgroup analyses further showed a reduced incidence of NPDR in the lower aDCSI score group and in the patient group for whom metformin was prescribed within three months after T2DM was diagnosed. Overall, these findings reemphasize the need for good glycemic control and earlier introduction of antidiabetic therapy if the aim of treatment is to minimize the risk of NPDR [43, 44].

### 4.1. Strengths and Limitations

A major strength of this study was its highly representative, population-based design within a universal health care system with nearly complete outpatient and inpatient visit information and prescriptions, leaving little room for selection bias. The limitations of our study include the use of an administrative database that lacks records of patient lifestyles and laboratory data, such as smoking, body mass index, and hemoglobin A1c, which may be considered confounding factors. Moreover, we were not certain whether patients had taken all of their antidiabetic medications as prescribed, and we also lacked information on patients' over-the-counter drug use. Finally, some metformin nonusers with higher aDCSI scores were excluded following propensity score matching. Therefore, analysis of unmatched population might have exaggerated the metformin effect toward a greater benefit than that recorded in our matched cohort. Although randomized, controlled studies are needed to confirm or refute the present findings, attempting to conduct clinical trials to test the effect of metformin in patients with T2DM and severe retinopathy are principally impractical, labor intensive, and very time-consuming. Hence, high-quality observational data might be the strongest type of evidence available for this kind of study.

## 5. Conclusion

Our study identified an inverse association between metformin therapy and the risk of NPDR among T2DM patients, and a potential trend was found for a reduced STDR risk in patients who had previously been diagnosed with NPDR. Combination therapy of metformin with DPP-4i seemingly had a significant beneficial effect against NPDR risk, especially when aDCSI scores were low and when metformin was prescribed as early as possible after T2DM diagnosis. Clinical management of DR presents a growing challenge because of the rising global epidemic of T2DM. Improving therapies to prevent DR would be particularly useful to treat the asymptomatic early stages of the disease. Protocols including metformin could optimally serve this purpose due to the well-established safety profile and efficacy of this drug during the initial treatment of T2DM.

## Figures and Tables

**Table 1:** Baseline characteristics of metformin users and nonusers with diabetes mellitus.

	Prepropensity							Postpropensity						
	Total (*N* = 29,638)		Metformin user (*N* = 24,611)		Metformin nonuser (*N* = 5,027)		*p* value	Total (*N* = 10,044)		Metformin user (*N* = 5,022)		Metformin nonuser (*N* = 5,022)		*p* value
	*N*	%	*N*	%	*N*	%		*N*	%	*N*	%	*N*	%	
Age (years)														
<45	5,989	20.21	5,263	21.38	726	14.44	<0.0001	1,449	14.43	723	14.40	726	14.46	0.9977
45–54	9,164	30.92	7,937	32.25	1,227	24.41		2,461	24.50	1,234	24.57	1,227	24.43	
55–64	7,509	25.34	6,313	25.65	1,196	23.79		2,386	23.76	1,190	23.70	1,196	23.82	
≥65	6,976	23.54	5,098	20.71	1,878	37.36		3,748	37.32	1,875	37.34	1,873	37.30	
Gender														
Female	13,363	45.09	11,113	45.15	2,250	44.76	0.6068	4,501	44.81	2,251	44.82	2,250	44.80	0.9840
Male	16,275	54.91	13,498	54.85	2,777	55.24		5,543	55.19	2,771	55.18	2,772	55.20	
Comorbidity														
Hypertension	11,809	39.84	9,460	38.44	2,349	46.73	<0.0001	4,699	46.78	2,355	46.89	2,344	46.67	0.8259
Cerebrovascular accidents (CVA)	14,398	48.58	11,530	46.85	2,868	57.05	<0.0001	5,744	57.19	2,881	57.37	2,863	57.01	0.7166
Diabetic polyneuropathy (DPN)	17	0.06	11	0.04	6	0.12	0.0439	9	0.09	4	0.08	5	0.10	0.7388
Diabetic nephropathy (DN)	76	0.26	57	0.23	19	0.38	0.0615	27	0.27	10	0.20	17	0.34	0.1773
Dyslipidemia	3,439	11.60	2,805	11.40	634	12.61	0.0143	1,265	12.59	631	12.56	634	12.62	0.9281
Medication														
Antihypertensive drugs	19,197	64.77	15,557	63.21	3,640	72.41	<0.0001	7,287	72.55	3,652	72.72	3,635	72.38	0.7039
Antihyperlipidemic drugs	10,113	34.12	8,293	33.70	1,820	36.20	0.0006	3,649	36.33	1,829	36.42	1,820	36.24	0.8519
aDCSI scores														
0	20,316	68.55	17,365	70.56	2,951	58.70	<0.0001	5,908	58.82	2,957	58.88	2,951	58.76	0.9916
1	4,528	15.28	3,694	15.01	834	16.59		1,665	16.58	832	16.57	833	16.59	
≥2	4,794	16.18	3,552	14.43	1,242	24.71		2,471	24.60	1,233	24.55	1,238	24.65	
DM duration^∗^ (years)														
0	21,639	73.01	18,148	73.74	3,491	69.44	<0.0001	6,979	69.48	3,488	69.45	3,491	69.51	0.9908
<4	5,890	19.87	4,830	19.63	1,060	21.09		2,113	21.04	1,056	21.03	1,057	21.05	
≥4 years	2,109	7.12	1,633	6.64	476	9.47		952	9.48	478	9.52	474	9.44	

^∗^Between DM diagnosis and antidiabetic drug prescription. aDCSI scores: adapted Diabetes Complications Severity Index scores; DM: diabetes mellitus.

**Table 2:** Risk of NPDR and STDR in patients with type 2 diabetes after propensity score matching.

	Metformin user events (total)	Metformin nonuser events (total)	Crude HR (95% CI)	Adjusted HR (95% CI)
NPDR	555/5022	578/5022	0.65 (0.58–0.73)	0.76 (0.68–0.87)
STDR	43/5022	68/5022	0.39 (0.27–0.58)	0.29 (0.19–0.45)
STDR among NPDR patients^∗^	43/860	14/182	0.50 (0.27–0.92)	0.54 (0.28–1.01)

^∗^NPDR patients were those who received any oral antidiabetic drugs after the diagnosis of NPDR among the study population. NPDR: nonproliferative diabetic retinopathy; STDR: sight-threatening diabetic retinopathy; HR: hazard ratio.

**Table 3:** Dose–response relation for risk of NPDR among DM patients after propensity score matching.

	DM patients without taking metformin	≤360 DDDs	361–720 DDDs	721–1080 DDDs	1081–1440 DDDs	>1440 DDDs
*N*	5022	2295	1312	637	357	421
NPDR (*n*, %)	578 (11.51)	262 (11.42)	162 (12.35)	70 (10.99)	29 (8.12)	32 (7.60)
NPDR onset time (years, mean ± SD)	3.61 ± 2.35	3.79 ± 2.40	5.28 ± 2.22	6.60 ± 2.21	7.69 ± 2.11	8.37 ± 1.97
Crude HR (95% CI)	Reference	0.95 (0.82–1.09)	0.73 (0.61–0.87)	0.53 (0.42–0.68)	0.33 (0.23–0.48)	0.27 (0.19–0.39)
Adjusted HR (95% CI)^∗^	Reference	0.99 (0.84–1.17)	0.77 (0.63–0.93)	0.53 (0.40–0.69)	0.33 (0.22–0.49)	0.29 (0.19–0.43)
*p* value		0.9115	0.0074	<0.0001	<0.0001	<0.0001
*p* for trend				0.0254^§^		
				0.0096^¶^		

^∗^Adjusted for gender, age, comorbidities, medications, aDCSI scores, DM duration, and other antidiabetic drugs use. ^§^Dose–response relation among DM patients. ^¶^Dose–response relation among DM patients with taking metformin. NPDR: nonproliferative diabetic retinopathy; DDD: defined daily dose; aDCSI scores: adapted Diabetes Complications Severity Index scores; DM: diabetes mellitus; HR: hazard ratio.

**Table 4:** Multivariate analysis for NPDR development in DM patients (*N* = 10,044).

	Model 1	Model 2	Model 3	Model 4	Model 5
	Crude HR (95% CI)	Adjusted HR^∗^ (95% CI)	Adjusted HR^§^ (95% CI)	Adjusted HR^¶^ (95% CI)	Adjusted HR^$^ (95% CI)
Sex					
Female	Reference	Reference	Reference	Reference	Reference
Male	0.84 (0.75–0.94)	0.81 (0.72–0.91)	0.80 (0.71–0.90)	0.80 (0.71–0.90)	0.80 (0.71–0.91)
Age (years)					
<45	1.26 (1.04–1.52)	1.24 (1.01–1.52)	1.30 (1.06–1.6)	1.32 (1.07–1.62)	1.25 (1.02–1.54)
45–54	1.59 (1.36–1.86)	1.62 (1.38–1.91)	1.68 (1.43–1.98)	1.70 (1.44–2.00)	1.63 (1.38–1.92)
55–64	1.50 (1.28–1.76)	1.54 (1.31–1.81)	1.56 (1.33–1.83)	1.57 (1.34–1.85)	1.53 (1.30–1.80)
≥65	Reference	Reference	Reference	Reference	Reference
Comorbidities					
Hypertension	0.88 (0.78–0.99)	1.20 (0.96–1.51)	1.18 (0.94–1.47)	1.18 (0.95–1.48)	1.19 (0.95–1.49)
Cerebrovascular accidents (CVA)	0.81 (0.72–0.92)	0.74 (0.58–0.94)	0.76 (0.60–0.96)	0.75 (0.59–0.95)	0.74 (0.59–0.94)
Diabetic polyneuropathy (DPN)	1.80 (1.08–2.99)	1.78 (1.07–2.97)	2.14 (1.28–3.58)	2.10 (1.26–3.50)	1.90 (1.14–3.16)
Diabetic nephropathy (DN)	1.19 (0.82–1.73)	1.14 (0.78–1.67)	1.20 (0.82–1.76)	1.19 (0.81–1.74)	1.14 (0.78–1.66)
Dyslipidemia	0.89 (0.73–1.08)	0.96 (0.78–1.17)	0.90 (0.74–1.10)	0.90 (0.73–1.10)	0.94 (0.77–1.16)
Medication					
Antihypertensive drugs	0.68 (0.58–0.79)	0.75 (0.63–0.90)	0.80 (0.67–0.95)	0.80 (0.67–0.95)	0.78 (0.65–0.93)
Antihyperlipidemic drugs	0.76 (0.68–0.86)	0.76 (0.67–0.86)	0.86 (0.76–0.98)	0.86 (0.76–0.97)	0.82 (0.72–0.93)
aDCSI score					
0	Reference	Reference	Reference	Reference	Reference
1	1.04 (0.89–1.22)	1.21 (1.02–1.43)	1.22 (1.03–1.45)	1.21 (1.02–1.44)	1.23 (1.04–1.45)
≥2	1.04 (0.90–1.19)	1.36 (1.16–1.60)	1.37 (1.17–1.61)	1.35 (1.15–1.59)	1.37 (1.17–1.62)
Metformin use					
Yes	0.65 (0.58–0.73)		0.76 (0.68–0.87)		
No	Reference		Reference		
Sulfonylurea					
Yes	1.28 (1.04–1.58)		1.30 (1.05–1.61)		
No	Reference		Reference		
Mitiglinide					
Yes	0.79 (0.67–0.92)		0.91 (0.78–1.06)		
No	Reference		Reference		
Acarbose					
Yes	0.74 (0.65–0.85)		0.87 (0.75–1.01)		
No	Reference		Reference		
TZD					
Yes	0.84 (0.73–0.97)		1.10 (0.94–1.28)		
No	Reference		Reference		
DPP-4i					
Yes	0.38 (0.31–0.48)		0.41 (0.33–0.52)		
No	Reference		Reference		
Metformin and DPP-4i use					
Neither metformin nor DPP-4i				Reference	
Metformin alone				0.76 (0.67–0.86)	
DPP-4i alone				0.36 (0.22–0.60)	
Both metformin and DPP-4i				0.32 (0.25–0.41)	
Metformin and sulfonylurea use					
Neither metformin nor sulfonylurea					Reference
Metformin alone					0.98 (0.65–1.46)
Sulfonylurea alone					1.56 (1.15–2.11)
Both metformin and sulfonylurea					1.02 (0.75–1.39)

^∗^Adjusted for gender, age at DM diagnosis, comorbidities, medication use, and aDCSI scores. ^§^Adjusted for gender, age at DM diagnosis, comorbidities, medication use, aDCSI scores, and antidiabetic drug use. ^¶^Adjusted for gender, age at DM diagnosis, comorbidities, medication use, aDCSI scores, and metformin or DPP-4i use. ^$^Adjusted for gender, age at DM diagnosis, comorbidities, medication use, aDCSI scores, and metformin or sulfonylurea use. NPDR: nonproliferative diabetic retinopathy; aDCSI scores: adapted Diabetes Complications Severity Index scores; DM: diabetes mellitus; HR: hazard ratio.

**Table 5:** Risk of NPDR in different metformin dose and aDCSI score groups (metformin+DPP4i vs. metformin alone).

	Total		<3 months		3 months to 1.5 years		>1.5 years	
	HR (95% CI)	Adjusted HR^∗^ (95% CI)	HR (95% CI)	Adjusted HR^∗^ (95% CI)	HR (95% CI)	Adjusted HR^∗^ (95% CI)	HR (95% CI)	Adjusted HR^∗^ (95% CI)
Metformin dose								
≤360 DDDs	0.50 (0.33–0.76)	0.49 (0.32–0.75)	0.37 (0.21–0.67)	0.38 (0.21–0.68)	0.48 (0.19–1.23)	0.46 (0.17–1.19)	1.37 (0.59–3.17)	1.34 (0.57–3.15)
361–720 DDDs	0.40 (0.25–0.64)	0.36 (0.22–0.59)	0.38 (0.20–0.74)	0.36 (0.19–0.69)	0.28 (0.09–0.94)	0.28 (0.08–0.96)	0.58 (0.24–1.42)	0.45 (0.18–1.15)
>720 DDDs	0.61 (0.41–0.9)	0.59 (0.39–0.88)	0.51 (0.31–0.85)	0.50 (0.3–0.83)	0.65 (0.28–1.49)	0.62 (0.26–1.44)	1.19 (0.39–3.65)	1.36 (0.41–4.49)
aDCSI score								
0	0.46 (0.34–0.62)	0.45 (0.33–0.61)	0.36 (0.24–0.54)	0.36 (0.24–0.55)	0.50 (0.25–0.97)	0.49 (0.24–0.97)	1.05 (0.51–2.17)	0.96 (0.46–2.02)
1	0.31 (0.16–0.58)	0.32 (0.17–0.60)	0.23 (0.09–0.58)	0.23 (0.09–0.58)	0.15 (0.02–1.09)	0.16 (0.02–1.18)	0.71 (0.26–1.96)	0.53 (0.18–1.58)
≥2	0.47 (0.24–0.91)	0.45 (0.23–0.89)	0.41 (0.18–0.95)	0.38 (0.16–0.89)	0.88 (0.18–4.36)	0.96 (0.17–5.35)	0.56 (0.12–2.54)	0.44 (0.09–2.13)

^∗^Adjusted for gender, age, comorbidities, medications, aDCSI scores, and other antidiabetic drugs use. NPDR: nonproliferative diabetic retinopathy; DDD: defined daily dose; aDCSI scores: adapted Diabetes Complications Severity Index scores; HR: hazard ratio.

## Data Availability

According to the Regulation of National Health Insurance Research Database (NHIRD) that the database shall not be provided for use by others outside the study and the computer host must be within the jurisdiction of the applicant's organization.

## Supplementary Materials

Supplementary Materials Supplementary Table 1: dose-response relation for risk of STDR among DM Patients after propensity score matching. Supplementary Table 2: risk of NPDR in different DDD levels of metformin and DDD4i groups (metformin+DPP-4i vs. metformin alone).
