# Supplementary material for: Metformin Treatment Is Associated with a Decreased Risk of Nonproliferative Diabetic Retinopathy in Patients with Type 2 Diabetes Mellitus: A Population-Based Cohort Study
Source: J Diabetes Res. 2020 Apr 19;2020:9161039. doi: 10.1155/2020/9161039 (PMC7189314; doi:10.1155/2020/9161039)
Supplement: Supplementary Materials — Supplementary Table 1: dose-response relation for risk of STDR among DM Patients after propensity score matching. Supplementary Table 2: risk of NPDR in different DDD levels of metformin and DDD4i groups (metformin+DPP-4i vs. metformin alone). [file 9161039.f1.zip › Supplementary_Table_1_0406.pdf]

Supplementary Table 1: Dose–Response relation for risk of STDR among DM Patients after propensity score matching

	DM patients without taking metformin	≤360 DDDs	361–720 DDDs	721–1080 DDDs	1081–1440 DDDs	>1440 DDDs
N	5022	2291	1310	639	357	425
STDR, n (%)	68 (1.35)	16 (0.70)	8 (0.61)	12 (1.88)	2 (0.56)	5 (1.18)
STDR onset time(years, mean ± SD)	5.31 ± 3.10	4.51 ± 2.49	5.08 ± 2.31	8.08 ± 1.79	10.6 ± 1.56	8.34 ± 1.05
Crude HR (95% CI)	Reference	0.52 (0.30–0.89)	0.31 (0.15–0.64)	0.67 (0.36–1.24)	0.15 (0.04–0.61)	0.25 (0.10–0.62)
Adjusted HR* (95% CI)	Reference	0.46 (0.26–0.81)	0.24 (0.11–0.52)	0.42 (0.21–0.84)	0.08 (0.02–0.33)	0.16 (0.06–0.44)
p value		0.0075	0.0004	0.0132	0.0006	0.0004

*adjusted for gender, age, comorbidities, medications, aDCSI scores, DM duration, and other antidiabetic drugs use

STDR, sight-threatening diabetic retinopathy; DDD, defined daily dose; aDCSI scores, adapted Diabetes Complications Severity Index scores; DM, diabetes mellitus; HR, hazard ratio; SD, standard deviation
