# Supplementary material for: Metformin Treatment Is Associated with a Decreased Risk of Nonproliferative Diabetic Retinopathy in Patients with Type 2 Diabetes Mellitus: A Population-Based Cohort Study
Source: J Diabetes Res. 2020 Apr 19;2020:9161039. doi: 10.1155/2020/9161039 (PMC7189314; doi:10.1155/2020/9161039)
Supplement: Supplementary Materials — Supplementary Table 1: dose-response relation for risk of STDR among DM Patients after propensity score matching. Supplementary Table 2: risk of NPDR in different DDD levels of metformin and DDD4i groups (metformin+DPP-4i vs. metformin alone). [file 9161039.f1.zip › Supplementary_Table_2_0406.pdf]

Supplementary Table 2: Risk of NPDR in different DDD levels of metformin and DDD4i groups (metformin + DPP-4i vs. metformin alone)

	Metformin dose ≤360 DDDs		Metformin dose 361–720 DDDs		Metformin dose >720 DDDs	
	HR (95% CI)	Adjusted HR* (95% CI)	HR (95% CI)	Adjusted HR* (95% CI)	HR (95% CI)	Adjusted HR* (95% CI)
Total						
DPP4i DDD= 0	Reference	Reference	Reference	Reference	Reference	Reference
0 < DPP4i DDD ≤ 150	0.52 (0.31–0.86)	0.50 (0.30–0.83)	0.51 (0.28–0.95)	0.47 (0.25–0.88)	0.75 (0.45–1.28)	0.76 (0.45–1.30)
DPP4i DDD > 150	0.46 (0.23–0.93)	0.48 (0.24–0.97)	0.30 (0.15–0.62)	0.29 (0.14–0.59)	0.51 (0.30–0.86)	0.46 (0.27–0.79)
Time to metformin use after DM diagnosis						
<3 months						
DPP-4i DDD= 0	Reference	Reference	Reference	Reference	Reference	Reference
0 < DPP-4i DDD ≤ 150	0.39 (0.19–0.79)	0.39 (0.19–0.80)	0.42 (0.17–1.03)	0.40 (0.16–1.00)	0.73 (0.39–1.39)	0.74 (0.39–1.42)
DPP-4i DDD > 150	0.35 (0.13–0.93)	0.35 (0.13–0.95)	0.36 (0.14–0.87)	0.33 (0.13–0.80)	0.38 (0.19–0.76)	0.36 (0.18–0.72)
3 months to 1.5 years						
DPP-4i DDD= 0	Reference	Reference	Reference	Reference	Reference	Reference
0 < DPP-4i DDD ≤ 150	0.53 (0.19–1.48)	0.50 (0.17–1.46)	0.33 (0.08–1.39)	0.32 (0.07–1.43)	0.93 (0.32–2.66)	0.84 (0.28–2.53)
DPP-4i DDD > 150	0.36 (0.05–2.63)	0.35 (0.05–2.54)	0.22 (0.03–1.65)	0.22 (0.03–1.65)	0.46 (0.14–1.53)	0.46 (0.14–1.54)
>1.5years						
DPP-4i DDD= 0	Reference	Reference	Reference	Reference	Reference	Reference
0 < DPP-4i DDD ≤ 150	1.45 (0.50–4.16)	1.72 (0.57–5.19)	1.09 (0.38–3.12)	0.80 (0.26–2.45)	0.50 (0.06–4.02)	0.53 (0.06–4.62)
DPP-4i DDD > 150	1.29 (0.39–4.26)	1.02 (0.29–3.57)	0.30 (0.07–1.28)	0.24 (0.06–1.05)	1.82 (0.55–6.04)	2.03 (0.58–7.04)

*adjusted for gender, age, comorbidities, medications, aDCSI scores, and other antidiabetic drugs use

NPDR, nonproliferative diabetic retinopathy; DDD, defined daily dose; aDCSI scores, adapted Diabetes Complications Severity Index scores; HR, hazard ratio;
